# ISTransbase: an online database for inhibitor and substrate of drug transporters

**DOI:** 10.1093/database/baae053

**Published:** 2024-06-29

**Authors:** Jinfu Peng, Jiacai Yi, Guoping Yang, Zhijun Huang, Dongsheng Cao

**Affiliations:** Xiangya School of Pharmaceutical Sciences, Central South University, No.172 Tongzipo Road, Changsha, Hunan 410031, China; Center of Clinical Pharmacology, The Third Xiangya Hospital, Central South University, No.138 Tongzipo Road, Changsha, Hunan 410031, China; School of Computer Science, National University of Defense Technology, No.869 Furong Middle Road, Changsha, Hunan 410073, China; Xiangya School of Pharmaceutical Sciences, Central South University, No.172 Tongzipo Road, Changsha, Hunan 410031, China; Center of Clinical Pharmacology, The Third Xiangya Hospital, Central South University, No.138 Tongzipo Road, Changsha, Hunan 410031, China; Center of Clinical Pharmacology, The Third Xiangya Hospital, Central South University, No.138 Tongzipo Road, Changsha, Hunan 410031, China; XiangYa School of Medicine, Central South University, No.172 Tongzipo Road, Changsha, Hunan 410031, China; Xiangya School of Pharmaceutical Sciences, Central South University, No.172 Tongzipo Road, Changsha, Hunan 410031, China

## Abstract

Drug transporters, integral membrane proteins found throughout the human body, play critical roles in physiological and biochemical processes through interactions with ligands, such as substrates and inhibitors. The extensive and disparate data on drug transporters complicate understanding their complex relationships with ligands. To address this challenge, it is essential to gather and summarize information on drug transporters, inhibitors and substrates, and simultaneously develop a comprehensive and user-friendly database. Current online resources often provide fragmented information and have limited coverage of drug transporter substrates and inhibitors, highlighting the need for a specialized, comprehensive and openly accessible database. ISTransbase addresses this gap by amassing a substantial amount of data from literature, government documents and open databases. It includes 16 528 inhibitors and 4465 substrates of 163 drug transporters from 18 different species, resulting in a total of 93 841 inhibitor records and 51 053 substrate records. ISTransbase provides detailed insights into drug transporters and their inhibitors/substrates, encompassing transporter and molecule structure, transporter function and distribution, as well as experimental methods and results from transport or inhibition experiments. Furthermore, ISTransbase offers three search strategies that allow users to retrieve drugs and transporters based on multiple selectable constraints, as well as perform checks for drug–drug interactions. Users can also browse and download data. In summary, ISTransbase (https://istransbase.scbdd.com/) serves as a valuable resource for accurately and efficiently accessing information on drug transporter inhibitors and substrates, aiding researchers in exploring drug transporter mechanisms and assisting clinicians in mitigating adverse drug reactions

**Database URL**: https://istransbase.scbdd.com/

## Introduction

Drug transporters, integral membrane proteins, play a crucial role in facilitating the transportation of substances across various tissues and organs in the human body. They are primarily categorized into two major superfamilies: the ATP-binding cassette (ABC) family and the solute carrier (SLC) family (1). These transporters engage in complex interactions with ligands, where a ligated one can function as both a substrate and an inhibitor for multiple drug transporters (2–4). Through their interactions with these ligands, drug transporters play essential roles in drug efflux and uptake, mediating drug–drug interactions (DDIs), and facilitating communication within cells, organs, interstitial fluids and even between organisms by interacting with endogenous substances (4, 5). Gaining a deep understanding of these intricate relationships is vital for unraveling the mechanisms of drug transport and comprehending their impact on therapeutic outcomes.

In the realm of drug discovery, research and development, a substantial amount of information is generated regarding drug transporters and their interactions with ligands. Health authorities, including the US Food and Drug Administration (FDA), the European Medicines Agency (EMA) and the Pharmaceuticals and Medical Devices Agency (PMDA), have recommended *in vitro* and *in vivo* approaches to assess the potential interactions between investigational drugs and transporters (6–8). Despite the availability of these guidelines and the abundance of data and materials generated through these methodologies, there is a lack of adequate collection, organization and efficient utilization of this wealth of information. Furthermore, a vast amount of valuable information is scattered throughout the scientific literature, hindering the attainment of a holistic understanding of drug transporter substrates and inhibitors. The scarcity of comprehensive data poses challenges for clinicians and researchers to swiftly and comprehensively understand the potential interactions and biological activities initiated by transporters. Consequently, there is an imperative need for an accessible and inclusive resource that can provide comprehensive insights into these complex interactions. Such a resource would greatly facilitate the work of clinicians and researchers by enabling them to access and utilize the available knowledge efficiently for drug development and clinical decision-making.

**Figure 1.**
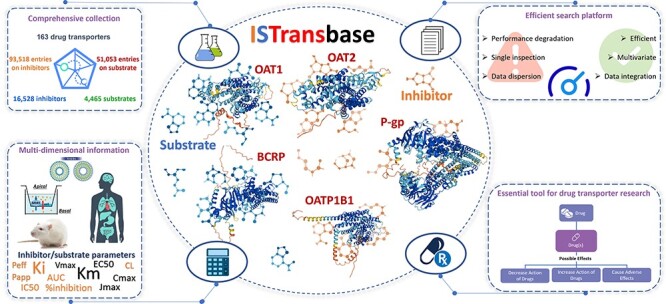
Graphical abstract of ISTransbase. ISTransbase provides comprehensive and detailed information on drug transporters and their inhibitors/substrates.

Computer databases serve as essential repositories of aggregated data records or files, enabling researchers and clinicians to efficiently access information on drug transporter substrates and inhibitors. Currently, several professional drug transporter databases are available, including UCSF-FDA (https://transportal.compbio.ucsf.edu/), Metrabase (https://www-metrabase.ch.cam.ac.uk/metrabaseui/), TP-Search (http://togodb.dbcls.jp/tpsearch) and VARIDT (http://varidt.idrblab.net/) (9–11). In addition, valuable information on transporters can also be obtained from databases such as DrugBank, PubChem and ChEMBL (12–14). However, these databases often suffer from scattered information on drug transporter inhibitors and substrates, alongside limitations in sample sizes or data completeness. To overcome these obstacles and streamline the identification of drug transporter inhibitors and substrates, we have developed the Inhibitor and Substrate of Transporters Database (ISTransbase, available at https://istransbase.scbdd.com/, Figure 1). ISTransbase offers comprehensive and detailed information on drug transporters substrates and inhibitors, as well as experimental methods, transport parameters, references, etc. By providing valuable tools for identifying inhibitors and substrates of drug transporters, ISTransbase aims to support drug development efforts and promote the judicious use of drugs by developers and clinicians. It serves as a valuable resource empowering users to explore and utilize extensive knowledge on drug transporter interactions, facilitating informed decision-making in drug development and clinical practice. By amalgamating and systematizing the dispersed information from diverse sources, ISTransbase stands as a pivotal advancement, offering a comprehensive and user-friendly platform for accessing critical data on drug transporter substrates and inhibitors.

## Materials and methods

### Data retrieval

To compile a comprehensive collection of inhibitors and substrates of drug transporters, an extensive search was conducted through multiple sources. This included literature searches, examination of government approval documents (such as those from the FDA and EMA) and reviewing guidelines from various Drugs Advisory Committees, such as the Anti-Infective Drugs Advisory Committee, Arthritis Drugs Advisory Committee, Bone, Reproductive and Urologic Drugs Advisory Committee, Pulmonary-Allergy Drugs Advisory Committee and Cardiovascular and Renal Drugs System N Drugs Advisory Committee. Specifically, our literature review targeted drug transporters, their inhibitors and substrates, employing databases such as PubMed, Web of Knowledge and Google Scholar, using a wide range of keywords, including ‘drug transporter’, and the specific names of over 80 transporters like MDR1/P-gp, BCRP, PEPT1, MRP1-4, OCT1, OATP1B1, OATP2B1, OATP1B3, BSEP, ASBT, etc.; ‘inhibitor’ and ‘substrate’ as additional keywords were used to refine our search. Furthermore, we accessed government approval documents from the FDA (Food and Drug Administration) and EMA (European Medicines Agency) websites, using URLs such as https://www.accessdata.fda.gov/scripts/cder/daf/index.cfm? event = BasicSearch.process, https://www.fda.gov/advisory-committees and https://www.ema.europa.eu/en/medicines. Python scripts were employed for text processing to extract preliminary information about drug transporters, their inhibitors and substrates. Data that clarified the relationship between drugs and transporters, specifying whether a drug acted as an inhibitor or substrate, were included in our dataset. This was followed by a rigorous manual review by another author to guarantee the data’s accuracy and reliability. This comprehensive approach enabled us to assemble a robust dataset of drug–transporter interactions, offering valuable insights into the roles of various transporters as inhibitors or substrates. Additionally, transporter substrates and inhibitors were searched in databases such as VIDART, UCSF-FDA, Metrabase, TP-Search, DrugBank, PubChem and ChEMBL. To ensure accuracy and specificity in identifying drug transporters, the TCDB database was referenced for transporter names and types (15).

### Data collection and processing

The collected information encompasses essential details, including transporter names, ligand types (inhibitor or substrate), experimental methods (*in vitro*/*in vivo*), treatment details (doses, concentrations, etc.) and experimental results (inhibition or transport parameters, values and units). Furthermore, ISTransbase stands as an invaluable asset, offering detailed annotations for every transporter protein. These annotations cover gene information, synonyms, structures, biological functions and significant polymorphisms extracted from sources such as UniProt (16), PDB (17), TCDB and SOLVO (https://www.solvobiotech.com/knowledge-center/transporters-a-z). In addition, both inhibitors and substrates are meticulously annotated with drug names, International Union of Pure and Applied Chemistry (IUPAC) names, Simplified Molecular-Input Line-Entry System (SMILES) notations, ligand types (inhibitor or substrate) and method types (*in vitro* or *in vivo*). This methodical approach guarantees that ISTransbase furnishes a rich repository of annotated data, facilitating them to access and explore detailed data on drug transporters, their ligands and associated experimental information.

The data of ISTransbase are stored in a database, primarily consisting of three tables: information tables for drugs and transporters, and a relationship table linking the two, where the relationship table connects the other two parts of information through foreign keys. During data retrieval, join operations are used to acquire all relevant data. For instance, when a user inputs the drug Cimetidine, the system retrieves all drug–transporter information related to this drug from the database, and through table join techniques, obtains detailed information on the corresponding transporter and drug.

### Online database implementation

The ISTransbase database has been successfully implemented and is currently hosted on an Elastic Compute Service (ECS) server, as previously mentioned (18, 19). The allocation of CPU cores and memory for running instances is dynamically adjusted based on demand, ensuring elastic computing capabilities. To ensure the longevity and reliability of the database, a long-term support strategy has been established to maintain and update the data regularly.

ISTransbase was engineered using the Python web framework Django 2.2. The web interface was created using Bootstrap 5.0 and SemanticUI 2.4, utilizing HTML5, CSS and JavaScript. MySQL was selected as the storage engine due to its balance between data capacity and query efficiency. The Nginx + uWSGI architecture was adopted to facilitate efficient data interchange between the dynamic server-side data and the static client-side content. For molecule visualization, RDKit was utilized to generate 2D images, while 3Dmol.js was employed to display the 3D structures of the transporters. All online data visualizations, including pie charts for search results, as well as bar charts and pie charts for statistical data, were implemented using ECharts 5.1. This open-source JavaScript library supports the swift creation of interactive visual displays. The website underwent thorough testing on various operating systems and web browsers to ensure compatibility and a seamless user experience. These measures were implemented to guarantee the reliability, functionality and user-friendliness of ISTransbase.

## Results

### Statistics in ISTransbase

**Figure 2.**
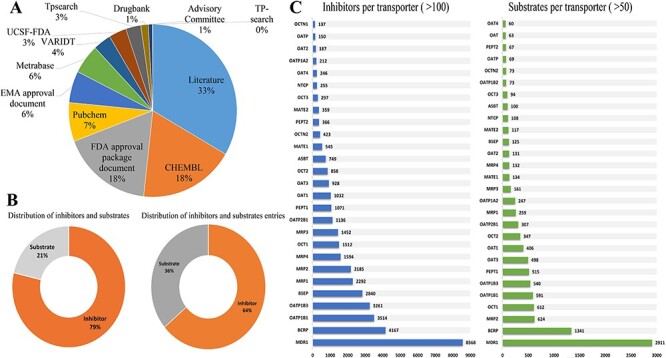
Statistics on the inhibitors and substrates of transporters. (A) Distribution of data sources in ISTransbase. (B) Distribution of inhibitors and substrates, and their entries of ISTransbase. (C) Inhibitors and substrates per transporters. Data were sourced from literature (33%), government reports (25%) and open databases (A). The database includes a comprehensive list of transporter inhibitors and substrates (B), covering 163 transporters from 18 species. This includes 17 ATP-binding cassette (ABC) transporters and 132 solute carrier (SLC) transporters (C).

ISTransbase is a comprehensive and meticulously curated database, offering extensive information on transporter inhibitors and substrates. The data collection process involved extensive research across literature, government documents and various open databases. Literature emerged as the primary data source, constituting 33% of the total, followed by multiple open databases like CHEMBL and PubChem, and government documents from FDA and EMA (Figure 2A).

The database contains an extensive collection of 16 528 inhibitors and 4465 substrates related to drug transporters, comprising 93 841 inhibitor entries and 51 053 substrate entries (Figure 2B). Of these, 83.88% of inhibitor entries and 73.55% of substrate entries are relevant to humans. The detailed distribution of human-related entries for each drug transporter is presented in Supplementary Table S1. ISTransbase covers a wide range of drug transporters, including 163 transporters from 18 different species. Among these transporters, 17 belong to the ATP-binding cassette (ABC) transporter family, including prominent ones such as Multidrug Resistance 1 (MDR1, P-gp, P-glycoprotein), Breast Cancer Resistance Protein (BCRP), Bile Salt Export Pump (BSEP) and Multidrug Resistance Proteins (MRPs). Additionally, there are 132 transporters from the solute carrier (SLC) transporter family, such as Organic Anion Transporting Polypeptides (OATPs), Organic Anion Transporters (OAT1/2/3/4), Organic Cation Transporters (OCT1/2/3) and Sodium Taurocholate Co-Transporting Polypeptides (NTCP). In the categorization of inhibitors and substrates by transporter, MDR1 stands out as the most significant for inhibitors, followed by BCRP, OATP1B1, OATP1B3 and BSEP (Table 1, Figure 2C).

**Table 1.** Amount of drug transporter inhibitors and substrates

	Inhibitor								Substrate							
	ISTransbase	UCSF	TP-Search	Metrabase	Drugbank	CHEMBL	Pubchem	VARIDT	ISTransbase	UCSF	TP-Search	Metrabase	Drugbank	CHEMBL	Pubchem	VARIDT
MDR1	8568	224	460	306	308	4591	2277	232	2911	45	314	566	360	641	101	427
BCRP	4167	107	63	616	116	1670	1054	123	1341	40	63	310	124	231	113	188
OATP1B1	3514	123	65	341	142	2090	152	246	591	45	41	97	61	80	22	89
OATP1B3	3261	72	10	260	72	2041	107	233	540	45	27	59	53	81	19	69
MRP1	2840	14	85	2	44	1469	605	39	125	11	61	98	27	77	27	48
BSEP	2292	632	40	NA	41	1179	1170	32	259	5	31	NA	72	31	4	7
MRP2	2185	612	98	161	38	659	701	67	624	18	84	160	51	96	27	106
OCT1	1594	189	85	291	57	296	165	232	132	42	49	166	37	98	34	82
MRP4	1512	628	54	NA	25	692	648	37	612	18	14	47	26	23	13	43
PEPT1	1452	6	108	274	36	375	318	44	161	9	31	247	26	66	71	59
MRP3	1136	622	34	48	15	650	621	19	307	13	17	65	13	22	6	28
OATP2B1	1071	92	36	136	48	254	20	234	515	15	26	48	23	60	11	36
OAT1	1032	149	152	NA	115	223	131	97	406	25	71	NA	53	70	21	62
OAT3	928	176	89	NA	91	130	68	103	498	36	56	NA	67	93	26	112
ASBT	858	7	23	11	8	331	301	9	347	5	9	54	5	8	4	6
OCT2	749	162	58	NA	76	115	94	96	100	48	35	NA	31	63	20	41
MATE1	545	111	1	NA	48	69	68	75	134	17	NA	NA	24	3	0	20
OCTN2	423	81	87	NA	47	96	3	16	73	15	14	NA	6	21	3	13
MATE2	366	86	1	NA	34	42	37	26	67	16	NA	NA	15	3	0	15
OCT3	359	45	32	NA	28	55	22	28	117	6	14	NA	13	19	8	13
PEPT2	297	8	91	NA	30	138	78	30	94	6	13	NA	12	14	1	24
NTCP	255	11	35	NA	15	52	29	18	108	7	28	NA	11	36	13	8
OAT4	246	42	48	NA	34	55	22	48	60	13	12	NA	8	13	8	13
OATP1A2	212	NA	37	50	53	37	2	29	247	13	29	56	29	32	15	52
OAT2	187	27	30	NA	21	30	10	38	131	11	20	NA	16	27	9	17
OCTN1	137	30	29	NA	23	30	0	11	43	12	6	NA	8	7	1	8
OATP1B2	10	NA	22	NA	NA	2	1	NA	73	NA	2	NA	NA	28	11	NA

Note: This table shows drug transporters with inhibitors >100 or substrates >30. Supporting information provides a brief explanation of the abbreviations for various transporters.

**Figure 3.**
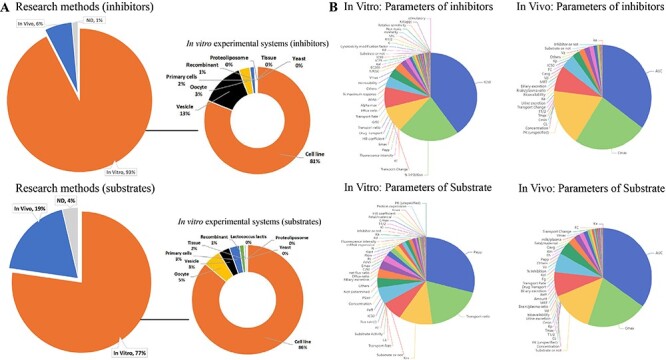
Statistics on research methods and parameters of transporter inhibitors and substrates. (A) Research methods and *in vitro* experimental system of inhibitor and substrate studies. (B) Distribution of *in vivo* and *in vitro* parameters of inhibitors and substrates. Analysis shows a higher prevalence of *in vitro* data for inhibitors (93%) and substrates compared to *in vivo* data (77%) (A). The most reported *in vitro* inhibition parameter is IC50 (40%). For substrates, the leading parameter is apparent permeability (Papp) (29%). *In vivo* studies mainly focus on the area under the concentration-time curve (AUC) for both inhibitors and substrates (B).

ISTransbase enables users to search for transporters related to drugs or drug-like compounds, providing valuable information such as transporter names, coding genes, structure, function, location in the body, associated diseases, significant polymorphisms and government policy information from FDA, EMA and PDMA. In addition to its comprehensive information on drug transporters, inhibitors and substrates, ISTransbase offers detailed insights into the experimental methodologies employed in both *in vivo* and *in vitro* studies. Analysis revealed a predominance of *in vitro* data, with cell lines being the primary experimental system for 81% of substrate research and 86% of inhibitor research (Figure 3A). Other commonly utilized experimental systems included membrane vesicles, oocytes and primary cells, among others.

ISTransbase has compiled the corresponding results obtained from these experiments, including transport parameters, inhibition parameters and qualitative findings. Among the *in vitro* inhibition parameters, the five most frequently reported were the drug dose causing 50% inhibition of drug transport (IC50) (40%), %inhibition (inhibition percentage of inhibitor) (21%), inhibition constant (Ki) (10%), transport change (8%) and half maximal effective concentration (EC50) (4%). For substrates, the most common parameters included apparent permeability (Papp) (29%), transport ratio (18%) and Michaelis constant (Km) (11%). In terms of *in vivo* studies, the predominant parameters for both inhibitors and substrates were the area under the concentration-time profile (AUC), maximum concentration (Cmax), clearance (CL) and concentration (Figure 3B). These parameters provide valuable insights into the pharmacokinetic behavior of the inhibitors and substrates.

### Search and download of ISTransbase

To optimize data retrieval in ISTransbase, we have implemented user-friendly searching and browsing tools, accessible through https://istransbase.scbdd.com/. The homepage features a search bar that allows users to query specific inhibitors, substrates or drug transporters, as well as perform drug–drug interaction checks. On the ‘Database’ page, users will find links that facilitate browsing and downloading of inhibitors or substrates according to their specific requirements. For additional assistance and information, the ‘About’ page provides detailed explanations of statistical information, tutorials and user terms and conditions.

#### Data searching

**Figure 4.**
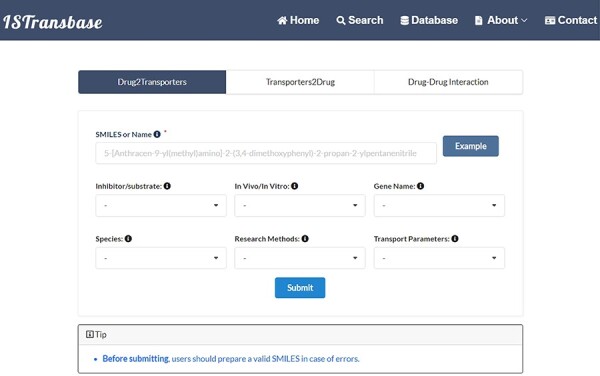
The main search page of ISTransbase. ISTransbase offers three search strategies that allow users to retrieve drugs and transporters based on multiple selectable constraints, as well as perform checks for drug–drug interactions.

ISTransbase offers three search modes (Figure 4) to facilitate data retrieval. The first, Drug2Transporters, enables users to search for drug transporters by entering the drug’s name or molecular information (SMILES) in the provided list box. The second mode, Transporter2Drugs, enables users to search for substrates and inhibitors associated with a specific drug transporter by selecting the transporter name or gene name from a drop-down list. Additional constraints, such as substrate/transporter, *in vivo*/*in vitro*, species, research methods and transport parameters, can be added simultaneously. The final mode, Drug-Drug Checker, assesses the potential for DDIs between inhibitors and substrates mediated by transporters. Selection of the ‘Submit’ button navigates users to the results page.

**Figure 5.**
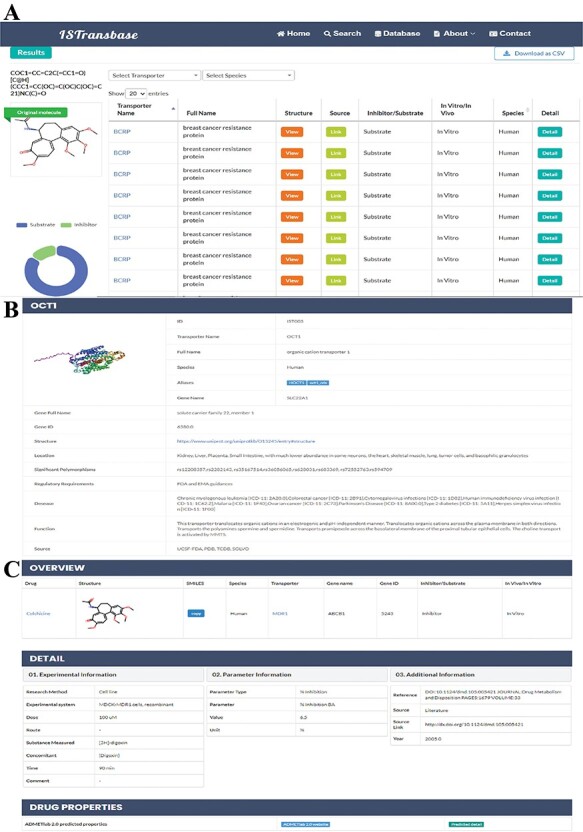
ISTransbase search result pages of Drug2Transporters mode. (A) Search results of drug2transporters mode (Colchicine: COC1 = CC = C2C(=CC1 = O)[C@H](CCC1 = CC(OC) = C(OC)C(OC) = C21)NC(C) = O). (B) Page with detailed information on drug transporters (OCT1). (C) Detailed view of Colchicine’s transport information mediated by the transporter MDR1.

On the Drug2Transporters results page, information is bifurcated into two main sections: the first provides essential molecule data and dynamic search statistics, detailing molecule names or SMILES, alongside a 2D structural image, and offers a statistical breakdown of search outcomes, categorizing ligand types, method types and species involved. The second section lists search results in a table, filter dropdown menu, downloadable in PDF or CSV formats, featuring transporter names, a ‘View’ option for transporter structure visualization, links to UniProt database entries, ligand and method types and species information (Figure 5A). Further, by selecting a transporter ID, users are directed to a comprehensive summary page for the transporter, encapsulating gene coding, structure, function, bodily location, associated diseases, significant polymorphisms and relevant governmental policy information (Figure 5B). The ‘Detail’ button opens a page divided into overview, experimental data and drug properties sections, offering in-depth insights into drug interactions, experimental methodologies, results (including parameters, values, units, references) and Absorption, Distribution, Metabolism, Excretion, and Toxicity (ADMET) properties predicted by ADMETlab 2.0 (20) (Figure 5C).

**Figure 6.**
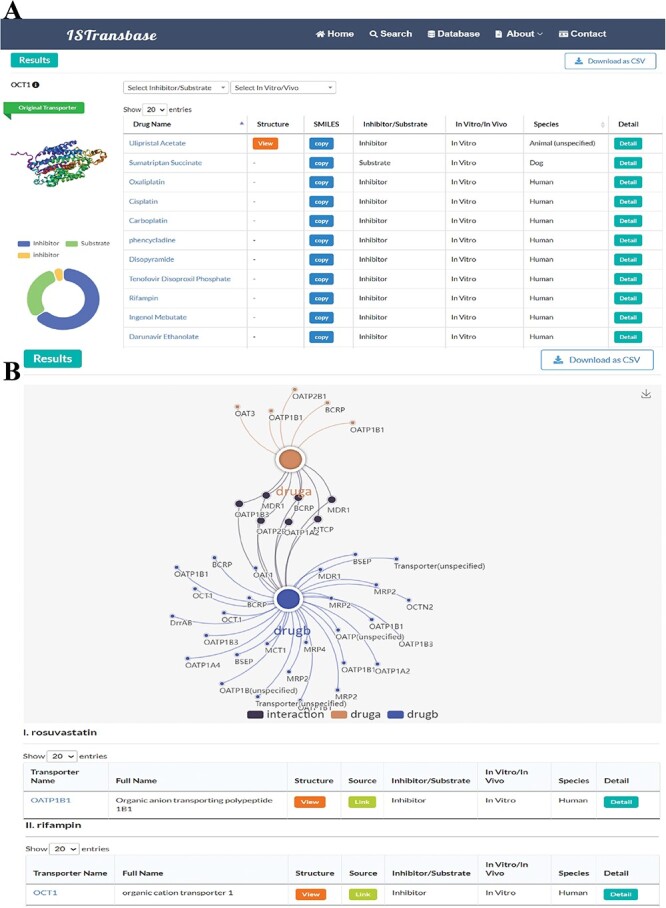
ISTransbase search result pages of Transporters2Drug and Drug-Drug Checker mode. (A) Search result of Transporters2Drug mode (OCT1). (B) Search result of Drug-Drug Checker mode (drug A: rosuvastatin, drug B: rifampin).

In the Transporters2Drug mode, users can search for inhibitors and substrates by selecting a specific transporter. The results are displayed on a similar page layout as described in the previous mode (Figure 6A). The left side displays the name and 3D structure of the transporter, as well as statistical results for inhibitors and substrates. The right side presents the details of the inhibitors and substrates in a table format. The table includes drug names that can be linked to DrugBank, molecular structures, SMILES for replication, ligand type (inhibitor/substrate), method type (*in vitro*/*in vivo*), species and a ‘Detail’ column that navigates to the detailed information page described earlier.

For the Drug-Drug Checker mode, when two drugs—referred to as drug A and drug B—are inputted using either their names or SMILES notation, ISTransbase creates a dynamic diagram. This diagram graphically depicts the drug transporters associated with these drugs. If drug A acts as an inhibitor and drug B as a substrate for the same transporter, it suggests potential for transporter-mediated drug interactions. Conversely, the scenario with drug A as a substrate and drug B as an inhibitor is similarly accounted for. In the diagram, these interactions are illustrated and distinguished by various colors, as demonstrated in Figure 6B. This methodology provides a clear, visual representation of possible transporter-mediated drug interactions, enabling easier analysis.

#### Data browsing and downloads

The browsing tool in ISTransbase provides a summary of the data by displaying the drug name, which is linked to Drugbank, the transporter name, the associated gene, the ligand type (inhibitor/substrate), the method type (*in vitro*/*in vivo*), the research methods used and a ‘Detail’ button for accessing more information. To assist users in refining their searches, ISTransbase offers filtering tools that allow for the selection of multiple criteria, including ligand type (inhibitor/substrate), method type (*in vitro*/*in vivo*), species, experimental system type and parameter type. Additionally, links to download the search results in Comma-separated values format are provided above the results. It is worth noting that all transporters, inhibitors and substrates can be downloaded from the website without the need for login or registration.

### Comparison with other resources

ISTransbase stands out as an extensive collection of drug transporter inhibitors and substrates, offering comprehensive and detailed information on transport and inhibition. While other databases such as UCSF-FDA, TP-Search, Metrabase, DrugBank, ChEMBL, Pubchem and VARIDT are available for searching drug transporter substrates and inhibitors (Table 1), they often lack comprehensive experimental data from authoritative sources like FDA and EMA, non-pharmaceutical molecules or endogenous substances. Such information would be invaluable for guiding clinical medication use, avoiding hazardous drug interactions and providing a reliable research methodology for transporter studies. Notably, databases such as DrugBank, ChEMBL and PubChem, despite housing a vast array of compounds, do not focus exclusively on transporter-related information and fall short in detailing precise methodologies and outcomes of transport and inhibition studies. This shortfall presents obstacles in the systematic aggregation of fragmented drug transporter data. In this context, ISTransbase emerges as the largest and most comprehensive database of drug transporter ligands (substrate and inhibition) available (Table 1).

Additionally, ISTransbase enhances research efficiency with its tri-modal search functionality, inclusive of multiple optional filters and direct links to external databases like DrugBank, UniProt and ADMETlab 2.0, thereby streamlining the identification process for drug transporter inhibitors and substrates.

## Discussion

Drug transporters play a crucial role in facilitating the transport of substances across cell membranes and interacting with their ligands, significantly impacting disease progression, pharmacokinetics, therapy and drug toxicity (21–23). Databases containing information on drug transporter inhibitors and substrates are invaluable resources for supporting pharmacokinetic studies, drug development and clinical applications. However, existing databases such as UCSF-FDA, Metrabase, Tp-search and VARIDT often have limited or insufficient data. These databases primarily focus on pharmaceutical molecules, potentially excluding non-pharmaceutical molecules and endogenous substances. ISTransbase addresses these gaps, providing a searchable, freely accessible compendium of detailed drug transporter information.

ISTransbase facilitates swift, comprehensive access to drug transporter data for clinicians and researchers, streamlining the compilation of information on drugs that are common substrates or inhibitors for multiple transporters across various organs. For instance, the transport of rosuvastatin involves intestinal OATP2B1 and BCRP (24, 25), hepatic OATPs (OATP 1B1, 1B3, and 2B1), NTCP and BCRP (3, 26–31), as well as renal OAT3 (32–34), and it can inhibit these transporters in reverse, posing risks like rhabdomyolysis (35, 36). ISTransbase serves as a centralized resource for accessing relevant data, aiding in the prevention of adverse effects and supporting scientific research, including the development of physiologically based pharmacokinetic models.

ISTransbase enables users to efficiently obtain a comprehensive understanding of DDIs mediated by drug transporters, as well as their associated methodologies. ISTransbase provides a significant amount of information on inhibitors and substrates for MRP1, MRP3, MRP4, PEPTs (human peptide transporters) and OATP2B1, suggesting their potential involvement in various drug–drug interactions. These drug transporters play critical roles in the development, treatment and resistance of diseases such as cancer, infections and cardiovascular diseases (37–39). Despite their current exclusion from national guidelines, they should be given proper consideration in drug development and clinical applications (6, 7, 40). Additionally, ISTransbase’s inclusion of literature on oocyte-based studies underscores the unique advantages of oocytes in transporter research, although further evaluation is needed to define their role in guidelines (41, 42).

While ISTransbase currently focuses on small molecule transporters, in the future, it will integrate data on biological macromolecules, such as nuclear receptors (43, 44) and nucleic acid molecules, including microRNAs (45, 46), in their interactions with drug transporters. These macromolecules have the potential to not only influence drug transporter activity but also regulate the expression of drug transporters. Meanwhile, we will continue our emphasis on transporter substrates and inhibitors documented in the existing literature, actively collaborating with government agencies to ensure the continuous updates of the database. Lastly, ISTransbase’s vast dataset paves the way for employing artificial intelligence to analyze transporter–ligand structural patterns, enhancing the prediction of transporter capabilities and inhibitor effects, thus contributing to the development of more accurate predictive models (47, 48).

## Conclusion

Based on its extensive database and user-friendly, efficient query system, ISTransbase can supply precise and comprehensive information regarding drug transporter substrates and inhibitors. This valuable resource aids clinicians and researchers in avoiding detrimental transporter-mediated interactions for patients, supports government departments in formulating drug transporter-related policies and facilitates the understanding of transporter mechanisms of operation, as well as the biological roles of its substrates and inhibitors.

## Supplementary Material

baae053_Supp

## Figures and Tables

**Table:** 

**Abbreviation**	**Definition**
%inhibition	Inhibition percentage of inhibitor
ABC	ATP-binding cassette
ADMET	Absorption, Distribution, Metabolism, Excretion, Toxicity
AUC	Area under the concentration-time profile
BCRP	Breast cancer resistance protein
BSEP	Bile salt export pump
CL	Clearance
Cmax	Maximum concentration
CPU	Central processing unit
DDI	Drug-drug interaction
EC50	Half maximal effective concentration
ECS	Elastic Compute Service
EMA	European Medicines Agency
FDA	US Food and Drug Administration
IC50	Drug dose causing 50% inhibition of drug transport or cell growth
IUPAC	International Union of Pure and Applied Chemistry
Ki	Inhibition constant
Km	Michaelis constant
MDR1	Multidrug Resistance 1
MRP	Multidrug Resistance Protein
NTCP	Sodium taurocholate cotransporting polypeptide
OAT	Organic anion transporter
OATP	Organic Anion Transporting Polypeptides
OCT	Organic cation transporter
Papp	Apparent permeability
PEPT	Human peptide transporter
PMDA	Pharmaceuticals and Medical Devices Agency
SLC	solute carrier
SMILES	Simplified molecular-input line-entry system

## Data Availability

ISTransbase is freely available at https://istransbase.scbdd.com/.
