# Supplementary material for: ISTransbase: an online database for inhibitor and substrate of drug transporters
Source: Database (Oxford). 2024 Jun 29;2024:baae053. doi: 10.1093/database/baae053 (PMC11214160; doi:10.1093/database/baae053)
Supplement: baae053_Supp [file baae053_supp.zip › suppl_data/Supporting Information.docx]

Supporting information for

**ISTransbase: An online database for inhibitor and substrate of drug transporters**

Jinfu Peng ^1 2^†*, Jiacai Yi ^4^†, Guoping Yang^1 2^, Zhijun Huang ^2 3^*, Dongsheng Cao ^1^*

1 Xiangya School of Pharmaceutical Sciences, Central South University, No.172 Tongzipo Road, Changsha, 410031, Hunan, China

2 Center of Clinical Pharmacology, The Third Xiangya Hospital, Central South University, No.138 Tongzipo Road, 410031, Changsha, Hunan, China

3 XiangYa School of Medicine, Central South University, No.172 Tongzipo Road, Changsha, 410031, Changsha, Hunan, China

4 School of Computer Science, National University of Defense Technology, No.869 Furong Middle Road, Changsha, 410073, Hunan, China

† Jinfu Peng and Jiacai Yi are equivalent authors to this work

* Correspondence: Tel: +86-731-89824761,[oriental-cds@163.com](mailto:oriental-cds@163.com); Tel.: +86-731-88618339, [huangzj@csu.edu.cn](mailto:huangzj@csu.edu.cn); Tel: +86-731-89824761, pengjinfu@csu.edu

**Table S1: Distribution of Human Species-Related Entries**

|  | **Substrate** | | | **Inhibitor** | | |
| --- | --- | --- | --- | --- | --- | --- |
| **Transporter** | **Entries of human** | **Total entries** | **Percentage of human (%)** | **Entries of human** | **Total entries** | **Percentage of human (%)** |
| MDR1 | 22552 | 28910 | 78.01 | 26598 | 31248 | 85.12 |
| BCRP | 3026 | 6119 | 49.45 | 8708 | 11427 | 76.21 |
| OATP1B1 | 1814 | 3140 | 57.77 | 5717 | 8031 | 71.19 |
| OATP1B3 | 1252 | 2248 | 55.69 | 4968 | 6519 | 76.21 |
| MRP2 | 1574 | 2715 | 57.97 | 3577 | 4785 | 74.75 |
| OCT1 | 1163 | 2212 | 52.58 | 2871 | 4944 | 58.07 |
| BSEP | 166 | 369 | 44.99 | 5047 | 6196 | 81.46 |
| OCT2 | 899 | 1538 | 58.45 | 2889 | 4484 | 64.43 |
| OAT3 | 853 | 1600 | 53.31 | 2039 | 3599 | 56.65 |
| OAT1 | 667 | 1266 | 52.69 | 2020 | 3834 | 52.69 |
| MRP1 | 475 | 648 | 73.30 | 3554 | 3690 | 96.31 |
| PEPT1 | 433 | 1186 | 36.51 | 1817 | 2590 | 70.15 |
| OATP2B1 | 550 | 975 | 56.41 | 1774 | 2377 | 74.63 |
| MRP4 | 40 | 194 | 20.62 | 2309 | 2514 | 91.85 |
| MRP3 | 96 | 347 | 27.67 | 1406 | 2345 | 59.96 |
| MATE1 | 402 | 612 | 65.69 | 1272 | 2005 | 63.44 |
| OCTN2 | 306 | 410 | 74.63 | 1259 | 1576 | 79.89 |
| MATE2 | 293 | 491 | 59.67 | 717 | 1246 | 57.54 |
| ASBT | 56 | 262 | 21.37 | 1055 | 1158 | 91.11 |
| OCT3 | 146 | 227 | 64.32 | 713 | 1037 | 68.76 |
| OATP1A2 | 525 | 702 | 74.79 | 247 | 408 | 60.54 |
| PEPT2 | 54 | 120 | 45.00 | 470 | 832 | 56.49 |
| OAT2 | 184 | 270 | 68.15 | 330 | 467 | 70.66 |
| NTCP | 131 | 281 | 46.62 | 327 | 446 | 73.32 |
| OAT4 | 82 | 97 | 84.54 | 485 | 574 | 84.49 |
| OCTN1 | 108 | 130 | 83.08 | 297 | 358 | 82.96 |

Note: This table shows drug transporters with total (inhibitor and substrate) entries > 400. The transporters marked in red are those recommended for investigation by the FDA, EMA or PMD.

**Table S2. Descriptions of Drug Transporters**

| **Abbreviation** | **Definition** | **Comment** |
| --- | --- | --- |
| MDR1 | Multidrug resistance 1 | The MDR1 gene codes for the production of a protein known as p-glycoprotein. This protein was originally discovered in cancer cells. During research, it was found that some lines of cancer cells are resistant to chemotherapy that is effective against other cancer cells. Further testing showed that these resistant cell lines contained a protein called p-glycoprotein, which sat on the cell membrane of these cancer cells and pumped chemotherapy drugs out of the cell. In this way, p-glycoprotein helped cancer cells resist the effects of chemotherapy drugs. |
| BCRP | Breast cancer resistance protein | This transporter functioned in both renal and extrarenal urate excretion. Plays a role in porphyrin homeostasis as it is able to mediates the export of protoporhyrin IX (PPIX) both from mitochondria to cytosol and from cytosol to extracellular space, and cellular export of hemin, and heme. Xenobiotic transporter that may play an important role in the exclusion of xenobiotics from the brain. Appears to play a major role in the multidrug resistance phenotype of several cancer cell lines. |
| OATP1B1 | Organic anion transporting polypeptides 1B1 | This transporter involved in the clearance of bile acids and organic anions from the liver. Mediates the Na(+)-independent uptake of organic anions such as pravastatin, taurocholate, methotrexate, dehydroepiandrosterone sulfate, 17-beta-glucuronosyl estradiol, estrone sulfate, prostaglandin E2, thromboxane B2, leukotriene C3, leukotriene E4, thyroxine and triiodothyronine. |
| OATP1B3 | Organic anion transporting polypeptides 1B3 | This transporter involved in the clearance of bile acids and organic anions from the liver. Mediates the Na(+)-independent uptake of organic anions such as 17-beta-glucuronosyl estradiol, taurocholate, triiodothyronine (T3), leukotriene C4, dehydroepiandrosterone sulfate (DHEAS), methotrexate and sulfobromophthalein (BSP). |
| MRP1 | Multidrug resistance protein 1 | This transporter mediates export of organic anions and drugs from the cytoplasm. Mediates ATP-dependent transport of glutathione and glutathione conjugates, leukotriene C4, estradiol-17-beta-o-glucuronide, methotrexate, antiviral drugs and other xenobiotics. Confers resistance to anticancer drugs. Hydrolyzes ATP with low efficiency. |
| BSEP | Bile salt export pump | This transporter involved in the ATP-dependent secretion of bile salts into the canaliculus of hepatocytes. |
| MRP2 | Multidrug resistance protein 2 | This transporter mediates hepatobiliary excretion of numerous organic anions and may function as a cellular cisplatin transporter. |
| OCT1 | Organic cation transporter 1 | This transporter translocates organic cations in an electrogenic and pH-independent manner. Translocates organic cations across the plasma membrane in both directions. Transports the polyamines spermine and spermidine. Transports pramipexole across the basolateral membrane of the proximal tubular epithelial cells. The choline transport is activated by MMTS. |
| MRP4 | Multidrug resistance protein 4 | This transporter may be an organic anion pump relevant to cellular detoxification. |
| PEPT1 | Human peptide transporter 1 | This tranporter mediates intake of oligopeptides of 2 to 4 amino acids with a preference for dipeptides and may constitute a major route for the absorption of protein digestion end-products. |
| MRP3 | Multidrug resistance protein 3 | This transporter may act as an inducible transporter in the biliary and intestinal excretion of organic anions. Acts as an alternative route for the export of bile acids and glucuronides from cholestatic hepatocytes. |
| OATP2B1 | Organic anion transporting polypeptides 2B1 | This Na(+)-independent transporter mediates the transport of organic anions such as taurocholate, the prostaglandins PGD2, PGE1, PGE2, leukotriene C4, thromboxane B2 and iloprost. |
| OAT1 | Organic anion transporter 1 | This transporter involved in the renal elimination of endogenous and exogenous organic anions. |
| OAT3 | Organic anion transporter 3 | This transporter may mediates the excretion of endogenous and exogenous organic anions, especially from the brain and kidney. Involved in the transport basolateral of steviol, fexofenadine. Transports benzylpenicillin (PCG), estrone-3-sulfate (E1S), cimetidine (CMD), 2,4-dichloro-phenoxyacetate (2,4-D), p-amino-hippurate (PAH), acyclovir (ACV) and ochratoxin (OTA). |
| ASBT | Apical sodium-dependent bile acid transporter | This transporter plays a critical role in the sodium-dependent reabsorption of bile acids from the lumen of the small intestine. |
| OCT2 | Organic cation transporter 2 | This transporter is a major determinant of the anticancer activity of oxaliplatin and may contribute to antitumor specificity. Mediates tubular uptake of organic compounds from circulation. |
| MATE1 | Multidrug and toxin extrusion protein 1 | This transporter is responsible for secretion of cationic drugs across the brush border membranes. |
| OCTN2 | Organic cation/carnitine transporter 2 | This sodium-ion dependent transporter involved in the active cellular uptake of carnitine. Transports one sodium ion with one molecule of carnitine. Also transports organic cations such as tetraethylammonium (TEA) without the involvement of sodium. |
| MATE2 | Multidrug and toxin extrusion protein 2 | This transporter is reesponsible for the secretion of cationic drugs across the brush border membranes, such as tetraethylammonium (TEA), 1-methyl-4-phenylpyridinium (MPP), cimetidine, N-methylnicotinamide, metformin, creatinine, guanidine, procainamide, topotecan, estrone sulfate, acyclovir, and ganciclovir. |
| OCT3 | Organic cation transporter 3 | This transporter may play a significant role in the disposition of cationic neurotoxins and neurotransmitters in the brain and mediate potential-dependent transport of a variety of organic cations. |
| PEPT2 | Peptide transporter 2 | This tranporter mediates intake of oligopeptides of 2 to 4 amino acids with a preference for dipeptides. Transports the dipeptide-like aminopeptidase inhibitor bestatin. Can also transport the aminocephalosporin antibiotic cefadroxil. |
| NTCP | Sodium/taurocholate cotransporting polypeptide | This tranporter strictly dependent on the extracellular presence of sodium and exhibits broad substrate specificity and transports various non-bile acid organic compounds as well. |
| OAT4 | Organic anion transporter 4 | This transporter mediates saturable uptake of estrone sulfate, dehydroepiandrosterone sulfate and related compounds. |
| OATP1A2 | Organic anion transporting polypeptides 1A2 | This Na(+)-independent transporter mediates the transport of organic anions such as sulfobromophthalein (BSP) and conjugated (taurocholate) and unconjugated (cholate) bile acids. Selectively inhibited by the naringin. |
| OAT2 | Organic anion transporter 2 | This sodium-independent transporter mediates the transport of multispecific organic anion. Transport of prostaglandin E2, prostaglandin F2, tetracycline, bumetanide, estrone sulfate, glutarate, dehydroepiandrosterone sulfate, allopurinol, 5-fluorouracil, paclitaxel, L-ascorbic acid, salicylate, ethotrexate, and alpha-ketoglutarate. |
| OCTN1 | Organic cation/carnitine transporter 1 | This sodium-ion dependent transporter mediates the tranport of carnitine and sodium-ion. Also transports organic cations such as tetraethylammonium (TEA) without the involvement of sodium. A key substrate of this transporter seems to be ergothioneine (ET). |
| OATP1B2 | Organic anion transporting polypeptides 1B2 | The organic anion-transporting polypeptide 1b family (Oatp1b2 in rodents and OATP1B1/1B3 in humans) is liver-specific and transports various chemicals into the liver. |
